# Proxies introduce bias in decoding TORC1 activity

**DOI:** 10.17912/micropub.biology.001170

**Published:** 2024-03-27

**Authors:** Marco Caligaris, Claudio De Virgilio

**Affiliations:** 1 Department of Biology, University of Fribourg, Fribourg, Fribourg, Switzerland

## Abstract

The eukaryotic TORC1 kinase integrates and links nutritional, energy, and hormonal signals to cell growth and homeostasis, and its deregulation is associated with human diseases including neurodegeneration, cancer, and metabolic syndrome. Quantification of TORC1 activities in various genetic settings and defined physiological conditions generally relies on the assessment of the phosphorylation level of residues in TORC1 targets. Here we show that two commonly used TORC1 effectors in yeast, namely Sch9 and Rps6, exhibit distinct phosphorylation patterns in response to rapamycin treatment or changes in nitrogen availability, indicating that the choice of TORC1 proxies introduces a bias in decoding TORC1 activity.

**Figure 1. Distinct phosphorylation patterns of Sch9 and Rps6 in response to dynamic TORC1 regulation:**
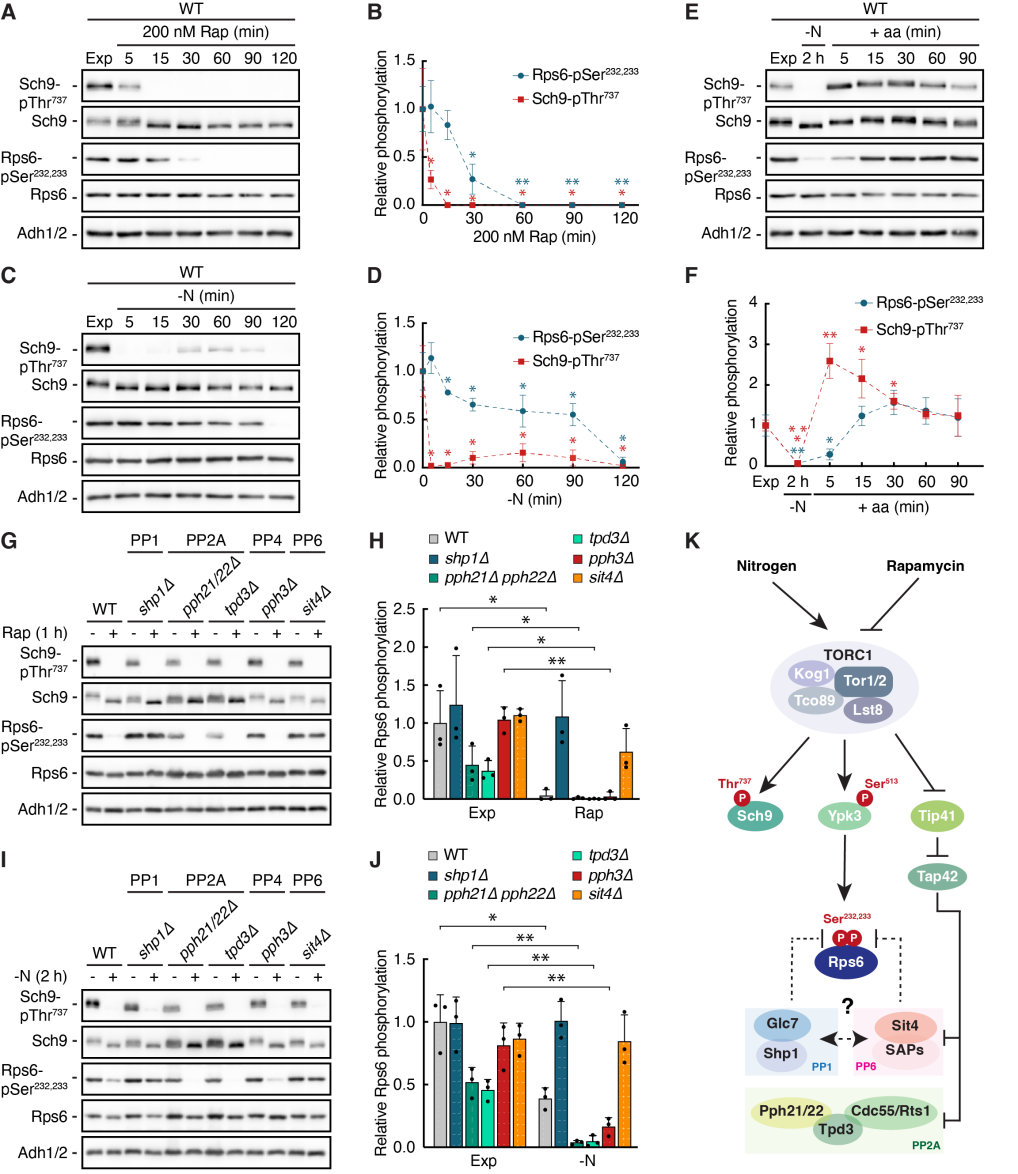
( **A, B** ) Prototrophic wild-type (WT) cells were grown exponentially (Exp) and treated with 200 nM rapamycin (Rap) for 5, 15, 30, 60, 90, and 120 min. Phosphorylations of the *bona fide* TORC1 target residue Sch9-Thr ^737^ and the Ypk3 target residues Rps6-Ser ^232,233^ were probed by immunoblot analyses of whole cell extracts using phospho-specific antibodies against the respective phospho-residues. Anti-Sch9 and anti-Rps6 antibodies served to detect the levels of Sch9 and Rps6, respectively. Adh1/2 levels probed with specific antibodies served as a loading control. (A). The mean TORC1 ( *i.e.* Sch9-pThr ^737^ /Sch9) and Ypk3 activities ( *i.e.* Rps6-pSer ^232,233^ /Rps6) were then quantified, normalized relative to the mean values of exponentially growing WT cells, and shown in (B) (n=3; ± SD; unpaired Student's t-test, *p≤0.05, **p≤0.005, ***p≤0.0005). ( **C, D** ) Prototrophic wild-type (WT) cells were grown exponentially (Exp), starved for nitrogen (-N) for 5, 15, 30, 60, 90, and 120 min, and analyzed (C) and quantified (D) as in (A) and (B), respectively. ( **E** ) Prototrophic wild-type (WT) cells were grown exponentially (Exp), starved for nitrogen (-N) for 2h, and then restimulated with a mix of all amino acids (+ aa) for 5, 15, 30, 60, and 90 min. Analyses (E) and quantifications (F) were performed as in (A) and (B), respectively. ( **G, H** ) Auxotrophic wild-type (WT), *shp1∆* , *pph21∆ pph22∆* , *tpd3∆* , *pph3∆* , and *sit4∆* cells were grown exponentially (Exp) and treated with 200 nM rapamycin (Rap) for 1 h (G) and analyzed as in (A). The mean Ypk3 activities were quantified, normalized relative to the mean value of exponentially growing WT cells, and shown in the bar diagram (H) (n=3; + SD; unpaired Student's t-test, *p≤0.05, **p≤0.005). ( **I, J** ) Auxotrophic wild-type (WT), *shp1∆* , *pph21∆ pph22∆* , *tpd3∆* , *pph3∆* , and *sit4∆* cells were grown exponentially (Exp), starved for nitrogen (-N) for 2 h (I), and analyzed as in (A). The mean Ypk3 activities (J) were quantified as in (H). ( **K** ) Model illustrating the key effector kinases and phosphatases emanating from TORC1 in budding yeast. TORC1 directly phosphorylates and activates Sch9 and Ypk3. A bona fide TORC1 target residue in Sch9 is Thr ^737^ in the hydrophobic motif (HM) (Urban et al., 2007), which is equivalent to Ser ^513^ in Ypk3 (González et al., 2015; Yerlikaya et al., 2016). Active Ypk3 phosphorylates Rps6 on Ser ^232,233^ (González et al., 2015; Yerlikaya et al., 2016). In parallel, TORC1 inhibits Tip41, an inhibitor of Tap42, which allows the latter to interact with and inhibit PP2A (Pph21/Pph22-Tpd3-Cdc55/Rts1) and PP6 (Sit4-SAPs). Dephosphorylation of Ser ^232,233^ in Rps6 in rapamycin-treated and nitrogen-starved cells requires both PP1 (Glc7-Shp1; (Yerlikaya *et al.* , 2016)) and, as shown here, PP6 (Sit4-SAPs). Arrows and bars refer to direct (full line) or indirect (dashed line) activating and inhibitory interactions, respectively. For details, see text.

## Description

The eukaryotic target of rapamycin complex 1 (TORC1) kinase is a central integrator of nutritional, energy, and hormonal signals that links these metabolic cues to cell growth and homeostasis. Genetically inherited or acquired deregulation of TORC1 uncouples growth and homeostasis from the respective signals, thereby establishing conditions that drive the emergence of human diseases such as neurodegeneration, epilepsy, immunodeficiencies, cancer, and metabolic syndrome (Albert and Hall, 2015; González and Hall, 2017; Laplante and Sabatini, 2012; Liu and Sabatini, 2020). Research in this field critically depends on the accurate quantification of TORC1 activities in various genetic settings and under defined physiological conditions. In mammalian cells, TORC1 activity is typically assessed via the phosphorylation levels of direct TORC1 target residues in the eukaryotic initiation factor 4E-binding protein 1 (4E-BP1; (Ma and Blenis, 2009) ) and the ribosomal protein S6 (rpS6) kinase 1 (S6K1) (Fenton and Gout, 2011; Magnuson et al., 2012) , or the ones in rpS6, the effector of the latter kinase (Meyuhas and Dreazen, 2009) . The most commonly used proxies for TORC1 activities in the budding yeast *Saccharomyces cerevisiae* include, similar to S6K phosphorylation in mammalian cells, phosphorylation of the *bona fide* TORC1 residue Thr ^737^ in the protein kinase Sch9 (Caligaris et al., 2023; Urban *et al.* , 2007) and, analogous to mammalian rpS6, phosphorylation of Ser ^232,233^ in Rps6, which is carried out by the Sch9-related TORC1 effector kinase Ypk3 (González *et al.* , 2015; Yerlikaya *et al.* , 2016). Accordingly, phospho-specific antibodies against Sch9-pThr ^737^ and Rps6-pSer ^232,233^ (that only recognize doubly phosphorylated Rps6) have been used to assess TORC1 activities under various physiological conditions such as limitation and starvation for amino acids or nitrogen and refeeding of amino acids or high-quality nitrogen sources to previously starved cells (Brito et al., 2019; Cecil et al., 2023; Chen et al., 2017; Chen et al., 2018; Hatakeyama et al., 2019; Liang et al., 2023; Péli-Gulli et al., 2015; Picazo et al., 2018; Takahara and Maeda, 2012; Vallejo et al., 2020). In these studies, both TORC1 proxies were used over highly disparate time lapses of treatment conditions ranging from minutes up to 6 hours (sometimes with intervals of minutes to hours). However, whether Sch9 and Rps6 phosphorylation can be used interchangeably to report TORC1 activity in yeast under these conditions is currently not known.

To address this question, we first compared the dynamics of Sch9-pThr ^737^ and Rps6-Ser ^232,233^ dephosphorylation in rapamycin-treated and nitrogen-starved cells. Following rapamycin treatment, Sch9-pThr ^737^ was very quickly dephosphorylated with a t _1/2_ of 2.6 min (95% CI = [1.34, 4.41] min), while Rps6-pSer ^232,233^ was dephosphorylated with much slower kinetics (t _1/2_ = 22.11 min; 95% CI [13.82, 41.08] min) ( Fig. 1A, B ). Interestingly, dephosphorylation of Sch9-pThr ^737^ was similarly swift upon nitrogen-starvation of cells, while Rps6-pSer ^232,233^ dephosphorylation did not even reach 50% after 90 min and only approached 0% after 2 h of nitrogen starvation ( Fig. 1C, D ). We speculate that the significantly delayed dephosphorylation of Rps6-pSer ^232,233^ may perhaps be due to limiting phosphatase activity as Rps6 is approximately 100-fold more abundant than Sch9 (SGD; (Breker et al., 2013; Chong et al., 2015; Ghaemmaghami et al., 2003) ). Alternatively, the different dephosphorylation kinetics of Sch9-pThr ^737^ and Rps6-pSer ^232,233^ may be explained by contrasting activities or substrate affinities of the respective phosphatases targeting these residues. Finally, it has also been proposed that TORC2 may in part phosphorylate Rps6-pSer ^232,233^ via the Ypk3-paralogs TORC2 effector kinases Ypk1/2, which may counteract the dephosphorylation of these residues when TORC2 remains active (Yerlikaya *et al.* , 2016). In sum, it appears that Rps6-pSer ^232,233^ dephosphorylation is a poor predictor of TORC1 inactivation when compared to Sch9-pThr ^737^ dephosphorylation specifically in cells that are starved for nitrogen for less than 90 min.

In parallel to the studies above, we have also compared the utility of Sch9 and Rps6 phosphorylation to detect TORC1 reactivation following addition of amino acids to nitrogen-starved cells. The phosphorylation of Sch9-Thr ^737^ was maximal after 5 min following amino acid re-addition, while the respective peak of Rps6-Ser ^232,233^ was delayed by about 15 min ( Fig. 1E, F ). Hence, both reporters can reveal TORC1 activation, but do so with different kinetics. The time lag in Rps6-Ser ^232,233^ phosphorylation may result from the fact that TORC1 favors Rps6 phosphorylation indirectly via Ypk3, or, again, be due to the high abundance of Rps6 or more effective counteracting phosphatase(s). Regarding the latter, we confirmed the previously published role of the type 1 protein phosphatase (PP1; Glc7) regulatory subunit Shp1 in the dephosphorylation of Rps6-pSer ^232,233^ in both rapamycin-treated and nitrogen-starved cells ( Fig. 1G -J) (Yerlikaya *et al.* , 2016; Zhang et al., 1995). Interestingly, however, we also discovered that the catalytic type 6 protein phosphatase (PP6) subunit Sit4 is equally important for this process, while the catalytic (Pph21 and Pph22) and scaffolding (Tpd3) subunits of the type 2A protein phosphatase (PP2A) and the catalytic (Pph3) subunit of the type 4 protein phosphatase (PP4) were not required for the dephosphorylation of Rps6-pSer ^232,233^ (Fig.1G-J) (Arino et al., 2019; Lillo et al., 2014). This suggests that either Glc7-Shp1 acts upstream of Sit4 (and the Sit4-associated [SAP] regulatory proteins) or *vice versa* . Alternatively, Glc7-Shp1 and Sit4-SAPs may each target only either pSer ^232^ or pSer ^233^ in Rps6 in a mutually exclusive, but partially cooperative way ( Fig. 1K ). Under the same conditions, dephosphorylation of pThr ^737^ in Sch9 remained unaffected by loss of the PP1 regulator Shp1, or by loss of PP2A (in *pph21∆ pph22∆* or *tpd3∆* cells), PP4 (in *pph3∆* cells), or PP6 (in *sit4∆* cells).

In conclusion, our data show that the assessment of TORC1 activity is strongly biased by the choice of the TORC1 target residues (direct such as Thr ^737^ in Sch9 or indirect such as Ser ^232,233^ in Rps6) that are probed for their phosphorylation levels. Accordingly, the relative affinities and activities of TORC1 and the counteracting protein phosphatases for a particular target residue, just as they are influenced by the relative abundance of these proteins, inevitably yield substrate-specific responses. Our data therefore align well with a more general paradigm shift in the TORC1 signaling field according to which TORC1 activity is not simply a uniform entity within a given cell, but an activity that can be locally (Betz and Hall, 2013; Hatakeyama *et al.* , 2019), temporally, and quantitatively harnessed for the phosphorylation of specific targets in response to discrete physiological cues (Cecil *et al.* , 2023; Nicastro et al., 2017; Powis and De Virgilio, 2016; Zeng et al., 2024).

## Methods

**Yeast strains and plasmids**

*Saccharomyces cerevisiae* strains and plasmids are listed in **Table 1** and **Table 2** , respectively. Gene deletions were performed using the pFA6a system-based PCR-toolbox (Janke et al., 2004) , and the primers listed in **Table 3** . Yeast cells were transformed via standard methods (Gietz and Woods, 2001) , as previously described (Deprez et al., 2023) . The transformation mix contained 240 μL 50% PEG, 36 μL 1 M LiAc, 5.3 µL of ssDNA (salmon sperm DNA solution), 15 µL of deletion cassette, and 54 µL of sterile H _2_ O. After the transformation, cells were washed 2 times with 1 mL of sterile H _2_ O and then plated on SD-Leu (synthetic dropout; 0.17% yeast nitrogen base, 0.5% ammonium sulfate [AS], 0.2% dropout mix without leucine [USBiological], and 2% glucose) plates. To identify the clones containing the correct deletion, colony PCR using the primers listed in **Table 3** was performed. Strains were rendered prototrophic, unless stated otherwise, by transforming them with the empty centromeric plasmids listed in **Table 2** . All strains and plasmids are available upon request.

**Growth conditions**

To maintain the plasmids, prototrophic cells were pre-grown in a synthetic dropout (SD; 0.17% yeast nitrogen base, 0.5% ammonium sulfate [AS], 0.2% dropout mix [USBiological], and 2% glucose) medium. Auxotrophic strains were pre-grown in a synthetic complete medium (SC; SD with all amino acids) medium. Subsequently, SC medium was used for the dilution of the cells the following day. When indicated, 200 nM rapamycin was added to the culture. Starvation experiments were performed by filtration and transfer of cells to a nitrogen starvation medium (0.17% yeast nitrogen base, 2% glucose, 0.01% adenine, and 0.005% uracil) for the indicated times. For amino acid re-addition experiments, a 25 times concentrated amino acid mix (25X dropout mix without histidine [USBiological] and 0.125% histidine) was added to the cultures, to reach the same concentration present in the SC medium. Cell growth was monitored by measuring the concentration (OD _600nm_ /mL) with a spectrophotometer.

**Cell lysate preparation and immunoblot analysis**

Cell lysates were prepared as described in (Hatakeyama *et al.* , 2019). Samples were denatured at 98°C for 5 minutes, loaded on SDS-PAGE, and transferred onto nitrocellulose membranes. Blocking with blocking buffer (5% milk powder in Tris-buffered saline) was performed for 1 h at room temperature. Membranes were immunoblotted with the primary antibodies listed in **Table 4** . After 3 washes, the membranes were incubated with the secondary antibodies conjugated with horseradish peroxidase listed in **Table 4** . Membranes were washed again 3 times and developed with ECL (GE Healthcare).

**Statistical analyses**

Three independent biological replicates of each experiment were performed. To determine the statistical significance, unpaired Student's t-test analysis was made with GraphPad Prism 10. Values with a p-value lower than 0.05 were considered significantly different. To express the variability, the standard deviation was calculated with GraphPad Prism 10 and shown in the graphs. In the figure legend, the number of independent replicas, the method used to express the variability, specific statistical tests, and significance are indicated.

## Reagents

**Table 1. Strains used in this study**

**Table:** 

**Strain**	**Genotype**	**Source**	**Panel**
BY4741	*MATa; ura3∆0, leu2∆0, his3∆1, met15∆0*	Euroscarf	G-J
YL515	[BY4741] *his3∆1, leu2∆0, ura3∆0*	(Binda et al., 2009)	A-F
MC380	[BY4741] *shp1∆::LEU2*	This study	G-J
YSB165-144-1C	[BY4741] *pph21∆::kanMX, pph22∆::kanMX*	CDV lab strain	G-J
YAL016w	[BY4741] *tpd3∆::kanMX*	Euroscarf	G-J
YDR075w	[BY4741] *pph3∆::kanMX*	Euroscarf	G-J
YDL047w	[BY4741] *sit4∆::kanMX*	Euroscarf	G-J

**Table 2. Plasmids used in this study**

**Table:** 

**Plasmid**	**Genotype**	**Source**	**Panel**
pRS413	* CEN, ARS, amp ^R^ , HIS3 *	(Brachmann et al., 1998)	A-F
pRS415	* CEN, ARS, amp ^R^ , LEU2 *	(Brachmann *et al.* , 1998)	A-F
pRS416	* CEN, ARS, amp ^R^ , URA3 *	(Brachmann *et al.* , 1998)	A-F
pFA-LEU2	* amp ^R^ , LEU2p-LEU2 *	This study	

**Table 3. Primers used in this study**

**Table:** 

**Name**	**Orientation**	**Sequence**
SHP1 pFA del For	Forward	TTTAAATATATAAGAAACGTCGGTAGCACAACAATTAACTCATTATTTAGGTATGCGGATCCCCGGGTTAATTAA
SHP1 pFA del Rev	Reverse	TTTATATATTAAGTTGAAGTCTTTTCCCGTTTCTGTTTTTGTATATTTATGCTCAGAATTCGAGCTCGTTTAAAC
SHP1 -253 For	Forward	AAGAAGCCAGCAAGTAGTGG
SHP1 +1483 Rev	Reverse	ATCACTTGGGGTGAATGCAG

**Table 4. Antibodies used in this study**

**Table:** 

**Name**	**Dilution**	**Source; product number**
Rabbit anti-ADH	1 :200000	Calbiochem; 126745
Rabbit anti-Sch9-phospho-Thr ^737^	1:10000	CDV lab
Goat anti-Sch9	1:1000	CDV lab
Rabbit anti-human-phospho-S6 ribosomal protein Ser ^235,236^	1:1000	Proteintech; 29223-1-AP
Guinea pig anti-Rps6	1:1000	(Yerlikaya *et al.* , 2016)
Goat anti-rabbit HRP conjugated	1:3000	BIO-RAD; 170-6515
Rabbit anti-goat HRP conjugated	1:3000	BIO-RAD; 5160-2104
Goat anti-guinea pig HRP conjugated	1:5000	Invitrogen; A18769
